# Trends in Web Searches About the Causes and Treatments of Autism Over the Past 15 Years: Exploratory Infodemiology Study

**DOI:** 10.2196/20913

**Published:** 2020-12-07

**Authors:** Florencia E Saposnik, Joelene F Huber

**Affiliations:** 1 Faculty of Social Sciences McMaster University Hamilton, ON Canada; 2 Paediatric Medicine The Hospital for Sick Children Toronto, ON Canada; 3 Division of Developmental Paediatrics Department of Paediatrics University of Toronto Toronto, ON Canada; 4 Surrey Place Toronto, ON Canada

**Keywords:** autism, infodemiology, infoveillance, informatics, Google Trends

## Abstract

**Background:**

Ninety percent of adults in the United States use the internet, and the majority of internet users report looking on the web for health information using search engines. The rising prevalence of autism spectrum disorder (ASD), uncertainty surrounding its etiology, and variety of intervention approaches contribute to questions about its causes and treatments. It is not known which terms people search most frequently about ASD and whether web search queries have changed over time. Infodemiology is an area of health informatics research using big data analytics to understand web search behavior.

**Objective:**

The objectives were to (1) use infodemiological data to analyze trends in web-based searches about the causes and treatments of ASD over time and (2) inform clinicians and ASD organizations about web queries regarding ASD.

**Methods:**

Google Trends was used to analyze web searches about the causes and treatments of ASD in the United States from 2004 to 2019. The search terms analyzed for queries about causes of ASD included vaccines, genetics, environmental factors, and microbiome and those for therapies included applied behavior analysis (ABA), gluten-free diet, chelation therapy, marijuana, probiotics, and stem cell therapy.

**Results:**

Google Trends results are normalized on a scale ranging from 0 to 100 to represent the frequency and relative interest of search topics. For searches about ASD causes, vaccines had the greatest frequency compared to other terms, with an initial search peak observed in 2008 (scaled score of 81), reaching the highest frequency in 2015 (scaled score of 100), and a current upward trend. In comparison, searches about genetics, environmental factors, and microbiome occurred less frequently. For web searches about ASD therapies, ABA consistently had a high frequency of search interest since 2004, reaching a maximum scaled score of 100 in 2019. The analyses of chelation therapy and gluten-free diet showed trending interest in 2005 (scaled score of 68) and 2007 (scaled score of 100), respectively, followed by a steady decline since (scaled scores of only 10 and 16, respectively, in 2019). Searches related to ASD and marijuana showed a rise in 2009 (scaled score of 35), and they continue to trend upward. Searches about probiotics and stem cell therapy have been relatively low (scaled scores of 22 and 18, respectively), but are gradually gaining interest. Web search volumes for stem cell therapy in 2019 surpassed both gluten-free diet and chelation therapy as web-searched interventions for ASD.

**Conclusions:**

Google Trends is an effective infodemiology tool to analyze large-scale web search trends about ASD. The results showed informative variation in search trends over 15 years. These data are useful to inform clinicians and organizations about web queries on topics related to ASD, identify knowledge gaps, and target web-based education and knowledge translation strategies.

## Introduction

The world wide web is often the first source of information people use to find answers about health [1]. Ninety percent of adults in the United States access the internet today [2], and the majority of internet users report looking for health information on the web in the past year [3]. Nearly 80% of those searching the web for health information begin their query using search engines, such as Google, rather than a dedicated health website to find information [3]. While the media has given rise to widespread information about autism spectrum disorder (ASD), it has also given rise to misinformation about ASD that has contributed to or perpetuated misperceptions, myths, and trends related to the causes and treatments of ASD [4]. Understanding lay beliefs, misconceptions, and queries about ASD can help identify areas that require public education and improved awareness [5], and analyzing web search behavior is one way to do this. This information can help direct clinician-patient discussions and can be used by ASD and health organizations to help guide web-based content and the development of web-based education strategies and knowledge translation plans.

Infodemiology is a relatively new field of scientific research and methodology in health informatics that involves the study of the distribution and determinants of web-based information, and infoveillance involves surveillance by tracking and analyzing trends in these data over time [6,7]. Web-based search analysis tools, like Google Trends, can be used to analyze data from large populations in order to understand web search behavior surrounding specific topics.

Google Trends is a big data web tool that applies algorithms to assess the frequency of web searches of large populations and compares and analyzes trends by stratifying the results based on geographic location, time period, category, and search type [6]. Google Trends data have proven to be useful in infodemiology research for analyzing human web search behavior about health topics [6]*.* Information gathered from infodemiology data for infoveillance, such as tracking a surge in web-based misinformation, can be used to identify the need for public health education strategies to counteract the misinformation and provide accurate and up-to-date information [6,7]. Analyzing web search patterns has been used to predict infectious outbreaks, track suicide trends, and identify specific periods of time for effective health promotion campaigns [8-10].

ASD is a complex neurodevelopmental disorder characterized by deficits in communication and social interaction, and restricted and repetitive patterns of behavior [11]. Individuals with ASD exhibit a varied spectrum of symptoms and severity, contributing to the complexity of understanding etiological factors. Although the etiology of ASD is still not fully understood and thought to be multifactorial, research suggests there is a strong genetic basis and/or predisposition in the majority of cases [12,13] and research continues to explore possible nongenetic contributing factors, such as environmental factors, and an interplay between genetic and environmental influences, which may either increase or reduce the risk of ASD [13].

The prevalence of ASD has markedly risen, and it is currently reported to occur in approximately 1 in 54 children [14]. While there is no singular treatment or cure for ASD, early and evidence-based interventions (eg, applied behavior analysis [ABA] strategies) with individualized treatment plans to target goals based on personal strengths, challenges, and needs have been shown to improve developmental outcomes [15].

The high prevalence and complexity of ASD along with the uncertainty surrounding its suspected multifactorial etiology have led to years of searching for answers about causes and looking for effective therapies [16]. While important advances through research have been made, many answers about etiology still remain elusive [16] and, perhaps more than with any other developmental disorder, there is controversy regarding the treatments of ASD [17]. While rigorous research continues to yield evidence-based information, there are many unfounded nonscientific approaches and myths surrounding the causes and treatments of ASD.

The web is a powerful tool for knowledge translation of health information, but it can also amplify alternative or partial truths and misinformation. It is not known which topics surrounding ASD causes and treatments are most frequently searched on the web. Google Trends data have been used to look at seasonal trends in web-based queries about mental health disorders, including ASD [18], and to evaluate the effectiveness of ASD awareness initiatives in promoting web searches on ASD [19]. To our knowledge, however, there are no infodemiology studies looking at trends in web-based queries about the causes and treatments of ASD and how web-based searches have changed over time.

The aim of this study was to use infodemiological data to identify and analyze trends in web-based queries about the causes and treatments of ASD and to evaluate how these trends have changed over the past 15 years. This information could help direct and enrich clinician-patient discussions and aid ASD advocacy and pediatric health organizations in developing web-based knowledge translation interventions and web-based ASD awareness campaigns and education strategies.

## Methods

### Methodology Framework

The general infodemiology and infoveillance methodology framework for Google Trends analyses, as outlined by Mavragani and Ochoa, was used [6]. This involves a four-step process. The Google Trends analysis tool tracks and quantifies the relative frequency of specific search terms on Google.

#### Identification of Search Terms

The first step is the identification of search terms. In this study, we selected “autism” instead of “autism spectrum disorder,” as it represents the most comprehensive word to identify ASD. The search term “autism” was combined with key search terms from questions about the *causes* and *treatments* of ASD commonly raised in the clinical setting.

##### Web Search Terms for the Causes of ASD

The terms selected for comparative Google Trends analyses for web queries about the causes of ASD were comprised of searches with the term “autism” with key words surrounding topics commonly covered by clinicians and questions commonly raised by parents or caregivers when discussing the cause of ASD. Further, these key words reflect topics and information covered by many ASD or pediatric health organization websites and social media groups. The search terms analyzed included “genetics,” “vaccines,” “environmental factors,” and “microbiome.”

##### Web Search Terms for the Treatment of ASD

The terms selected for Google Trends analyses for web queries about treatments for ASD were comprised of searches with the term “autism” with key words surrounding topics commonly covered by clinicians and questions commonly raised by parents or caregivers, when discussing intervention for ASD. These key words reflect well-established, popular, emerging, or speculative and/or alternative *therapies* for ASD and reflect topics and information covered by many ASD or pediatric health organization websites and social media groups. The search terms analyzed included “ABA” (applied behavior analysis), “gluten-free diet,” “chelation therapy,” “marijuana,” “probiotics,” and “stem cell therapy.” An additional analysis of the search term of fidget spinners (as they relate to ASD [“autism fidget spinner”]) was also performed in a separate Google Trends analysis, given their high popularity in the recent past and their popular association with disorders such as ASD. This term was analyzed separately as it is not necessarily viewed as an intervention in the same way as the other *intervention* search terms for ASD.

#### Geographical Region and Time Frame for Analyses

The second step involves selecting the geographical region where the data should be retrieved from, and the third step involves selecting the time frame over which the analyses are conducted. These analyses focused on Google searches in the United States over 15 years from 2004 (the earliest data available for Google Trends analysis) to 2019. Because the analyses spanned a very lengthy period of 15 years, with large amounts of data, the data were aggregated by year (rather than by day or month) for ease of interpretation using the Google Trends mobile app.

#### Google Trends Search Category for Analyses

The fourth step involves identifying a search category. Categories include options such as Arts & Entertainment, Finance, Food & Drink, Health, Sports, and News, in order to refine the search data analyses. Specifying a category helps to eliminate irrelevant data and to achieve more specific research results. The category of “Health” was used for all search term analyses.

### Google Trends Analyses

Using Google Trends, each data point is divided by the total searches made within the specified geography region over the specified time range to compare the relative popularity or the relative frequency of specific search terms. More specifically, the relative popularity or frequency of a search term is the ratio of a query’s search volume compared to the sum of the search volumes of all possible Google queries. Queries from the same web-based protocol address made over a short period of time are excluded from results by Google Trends analyses. The data are normalized on a scale ranging from 0 to 100 based on a search topic’s proportion to all searches, making the results comparable [6]. Google Trends algorithms were applied to assess and compare the frequency of the web search terms described, over the specified period (2004-2019) and geographical region (United States), and in the specified category of health.

## Results

### Web-Based Searches Related to the Causes of ASD

**Figure 1:**
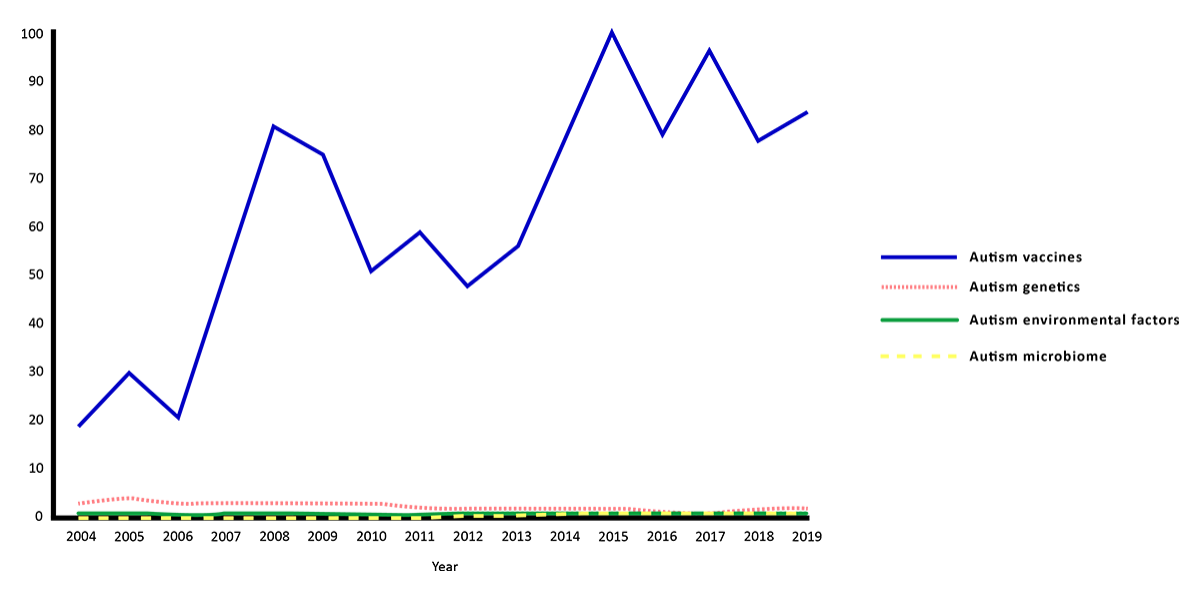
Google Trends analyses for web searches related to queries about the causes of autism in the United States from 2004 to 2019.

For web-based searches about the causes of ASD using the Google search engine (Figure 1), vaccines had the greatest search volumes when compared with other search terms, with an initial search peak observed in 2008 (scaled score of 81), reaching the maximum and highest search frequency in 2015 (scaled score of 100). There has been continued high interest in the following years, with a scaled score of 84 in 2019 and an upward trend in search interest since 2018 (scaled score of 78).

Google search queries about ASD and the role of known or proposed etiological factors, including genetics, environmental factors, and microbiome, occurred relatively and considerably less frequently compared to vaccines (scaled score of 84 in 2019), with minimal overall search interest over the past 15 years and scaled scores of 2, 1, and 1, respectively, in 2019.

### Web-Based Searches Related to Therapies for ASD

**Figure 2:**
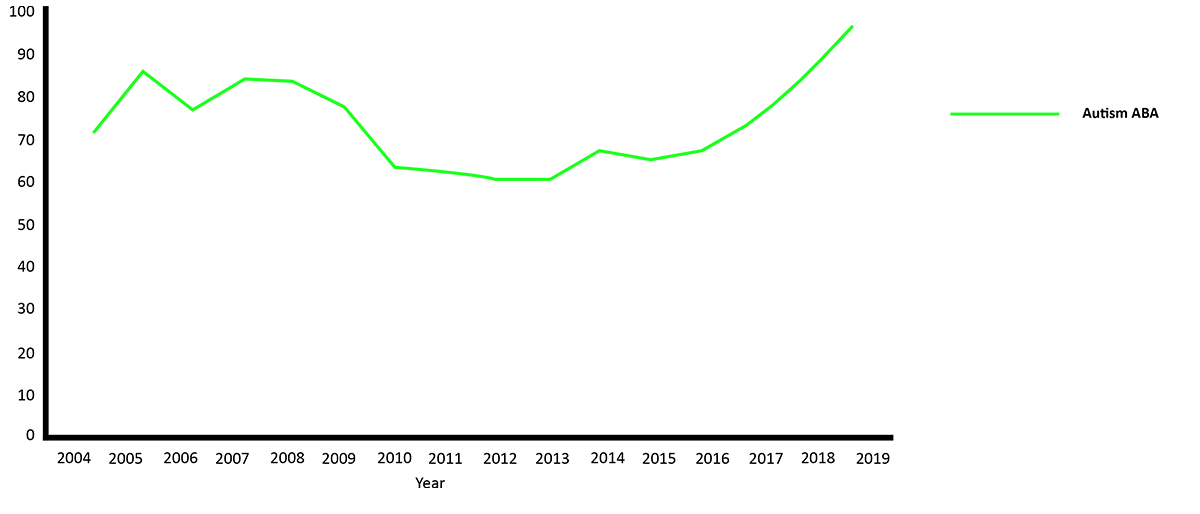
Google Trends analyses for web searches related to queries about applied behavior analysis (ABA) as an intervention for autism in the United States from 2004 to 2019.

For web-based searches about therapies for ASD using the Google search engine, ABA consistently had a high frequency of web search interest from 2004 to 2019 (Figure 2), with a slight decrease between 2010 and 2013, followed by a steady rise in search frequency, reaching a maximum scaled score of 100 in 2019.

**Figure 3:**
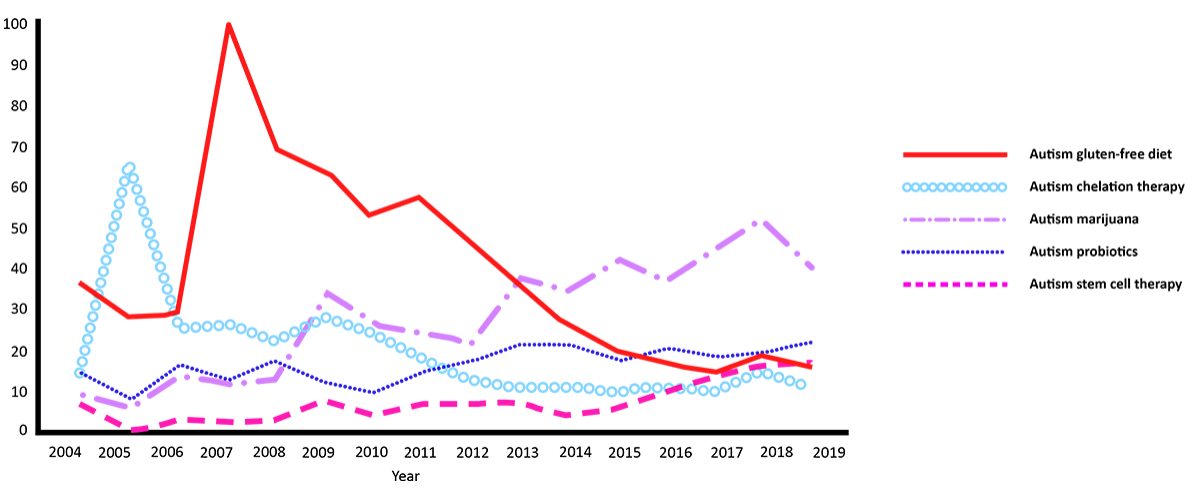
Google Trends analyses for web searches related to queries about interventions for autism in the United States from 2004 to 2019.

The analyses of ASD and chelation therapy (Figure 3) showed a precipitous high trending interest in 2005 (scaled score of 68 in 2005 compared to 14 in 2004), followed by a drop in search interest in 2006 (scaled score of 25) and an overall decline in search interest frequency in the following years, with a scaled score of only 10 in 2019.

The analyses of ASD and a gluten-free diet (Figure 3) showed rapid and high trending interest, with a maximum peak search frequency scaled score of 100 in 2007, followed by a steady overall decline in search interest frequency in the years following 2007 and a scaled score of only 16 in 2019.

While searches about ASD and probiotics (Figure 3) have been relatively low over the past 15 years compared to the frequency of other searches for ASD interventions, probiotics appear to be gradually gaining interest, with the highest scaled score over the past 15 years in 2019 (scaled score of 22). Search interest in probiotics and ASD surpassed the frequency of search interest in a gluten-free diet in 2016, which is a nutrition-based intervention that gained popular web search interest in 2007. Web search interest in probiotics and ASD has continued to be more popular than gluten-free diet searches since 2016.

Web searches related to ASD and marijuana (Figure 3) had an initial rise in interest in 2009 (scaled score of 35), possibly coinciding with the legalization and commercialization of marijuana in some jurisdictions [20], and continue to exhibit overall rising popularity in search interest. Apart from searches about ASD and ABA, which have consistently been high (Figure 2), marijuana and ASD have been searched much more frequently than all other intervention search terms evaluated since 2013 (Figure 3).

Web searches regarding ASD and stem cell therapy (Figure 3) have been relatively low, as they represent a topic of more recent research, but they are gradually gaining web search interest, with the highest search frequency in 2019 (scaled score of 18), surpassing the volume of web searches for both gluten-free diet and chelation therapy in 2019.

**Figure 4:**
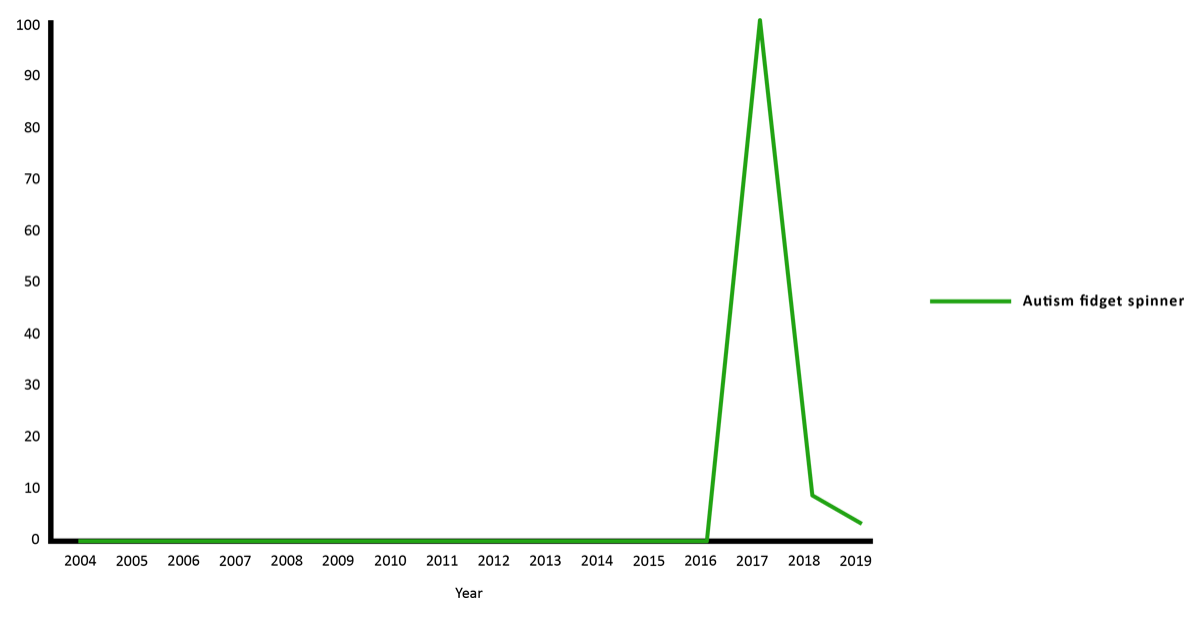
Google Trends analyses for web searches related to queries about autism and fidget spinners in the United States from 2004 to 2019.

Google Trends analyses for ASD and fidget spinners were performed separately (Figure 4) as fidget spinners fall into a unique category distinct from intervention or therapy in the traditional sense, but a common topic of interest has been whether they provide any benefits for children with ASD. ASD and fidget spinner searches had a scaled score of 0 prior to becoming popular in the market in 2017. In 2017, fidget spinner searches for ASD had a sudden maximum peak scaled score of 100, followed by a marked drop in search interest in the following 2 years, with scaled scores of 8 and 3 in 2018 and 2019, respectively (Figure 4).

## Discussion

### Principal Findings

Using Google Trends analyses is an effective infodemiology strategy to evaluate large-scale web search data about ASD. These analyses of web searches related to queries about the causes and treatments of ASD showed substantial, variegated, and informative patterns of changes in the frequency of web-based search interest over the past 15 years. Some topics emerged, trended, peaked, and then decreased in search frequency over time, while others remained highly stable topics of public interest demonstrated by a high frequency of web searches over time and still others showed low overall relative interest or are currently gaining public interest. These data provide valuable information and highlight the benefits of the ongoing use of web search analytic tools, as the information yielded can be used to inform clinicians as well as ASD and pediatric health organizations. These data can help guide clinical discussions, as well as education and knowledge translation strategies in order to provide up-to-date and accurate information on relevant topics to improve parent/caregiver, patient, and public awareness about ASD.

### ASD and Vaccines, and the Etiology of ASD

Our study confirms that despite the lack of evidence to support vaccines as a cause of ASD [21,22], this topic continues to be the most widely searched web-based topic regarding the cause of ASD on the Google search engine. The initial rise in web-search interest in ASD and vaccines occurred in 2007 and peaked in 2008. Some temporally related factors, which may have contributed to the rise in web searches about autism and vaccines during this time and beyond, were public statements about vaccines and ASD by celebrity Jenny McCarthy and the publication of her books [23,24]. Further, in 2008, Andrew Wakefield admitted to a disciplinary panel that he fabricated details [25] surrounding a study published in the Lancet in 1998 [26] insinuating that the measles, mumps, and rubella (MMR) vaccine may be linked to a risk of ASD. Despite its retraction [27], there was reduced uptake of vaccination and increased concern by parents regarding the vaccination, which was considered by some as a “public health crisis” and “perhaps, the most damaging medical hoax of the last 100 years” [28], and our study showed that relevant public web search interest on the topic of an autism-vaccine connection still continues today. In contrast, Google searches about the genetics of autism, which is believed to play an important role in the etiology of ASD, and possible environmental contributors [12,13,15,16], are relatively much less frequent. These data indicate that web-based content to increase public awareness and parent/caregiver and patient education surrounding the etiological factors of ASD, and vaccinations and ASD continue to be needed.

### ASD and ABA Therapy

ABA therapy is considered to be one of the most effective evidence-based interventions for ASD [15]. Our study revealed consistently high volumes of web-based searches over the past 15 years for information about ABA for ASD. This is encouraging as it indicates public awareness of ABA as an intervention for ASD and an interest in learning about this recommended intervention for children with ASD. Further, it highlights the need for ongoing discussions in the clinical setting and accessible web-based information about ABA.

### ASD and Chelation Therapy

Chelation therapy is a medical intervention that removes heavy metals from the body. It may be a treatment option to treat metal toxicity due to over exposure or to treat the accumulation of metals, such as iron and copper, that accumulate in the body due to certain medical conditions or diseases. It should only be administered by competent medical professionals. While not recommended as an intervention for ASD [29], it has been used by some as an alternative treatment for ASD [30], based on the unsubstantiated belief that ASD is caused by mercury or thimerosal exposure from vaccination [21]. Thimerosal is a mercury-based preservative that has been used in some vaccinations. According to the Centers for Disease Control and Prevention, the MMR vaccine has never contained thimerosal or mercury [31]. In 1999, it was recommended by the United States Public Health Service that thimerosal be removed as a preservative from childhood vaccines, as a precautionary measure, and “today, no childhood vaccine used in the US, except some formulations of flu vaccine in multidose vials, use thimerosal as a preservative” [31].

Numerous scientific research studies have shown no causal connection between exposure to thimerosal in vaccines and ASD [32-39]. Further, the diagnostic rates of ASD have continued to rise following the precautionary removal of thimerosal from childhood vaccinations [14]. Using chelation therapy as a treatment for children with ASD is not recommended and is in fact considered dangerous as it may lead to serious, harmful, and potentially deadly side effects [17,29].

This study showed that web searches for information about chelation therapy and ASD, which could yield misinformation about its benefits, precipitously rose in 2005, followed by a drop in search interest in 2006 and an overall decline in search interest frequency in the following years, with the lowest web search volumes of all ASD therapies studied. This result is reassuring, as it may reflect a decrease in interest in pursuing this dangerous and ineffective treatment for ASD.

### ASD and Nutrition and Gastroenterology

Research suggests that gastrointestinal disturbances are common among children with ASD [40]. This has led to interest in investigating the gut microbiome and a hypothesized microbiota-gut-brain axis in children with ASD [41-43]. Some preliminary investigations have suggested that there may be an imbalance of gut bacteria in some children with ASD [44]. Some researchers have suggested that by alleviating the gastrointestinal response, there could be a behavioral improvement in children with ASD [42]. This study showed that while there is currently a relatively low volume of web-based searches for information about the role of the gut microbiome and ASD, Google searches for information about the use of probiotics for individuals with ASD are on the rise. While some research may suggest that probiotics may have potential for treating gastrointestinal distress in some children, the evidence at this point does not support probiotics as the mainstay of treatment to be used indiscriminately in children with ASD [45,46].

Another alternative nutrition-based intervention that has been commonly linked with ASD is a gluten-free diet [29]. A gluten-free diet is a well-established treatment for individuals with celiac disease, a disorder involving a severe gastrointestinal response to foods containing gluten [47]. However, despite the popular use of a gluten-free diet by parents/caregivers of children with ASD, there is insufficient evidence to indiscriminately recommended it as a treatment for all children diagnosed with ASD [29,48,49]. Web search interest for a gluten-free diet and ASD had gained popularity, with a rapid and high trend, reaching a maximum peak search frequency in 2007. This was however followed by a steady overall decline in web-based search interest frequency in the years following, with relatively low search interest in recent years compared to other ASD interventions. Search interest in probiotics surpassed the frequency of search interest in a gluten-free diet in 2016 and has continued to be a more popular nutrition search term with ASD than a gluten-free diet since 2016. Parents/caregivers of children with ASD are encouraged to discuss their children’s individual nutritional needs with their physicians, as each child’s health, gastrointestinal presentation, and nutritional needs are different.

### ASD and Medicinal Marijuana/Cannabis

With changes in legislation and the commercialization of marijuana/cannabis in many regions in North America, there is rising interest in the use of medicinal marijuana for the treatment of various health conditions, including ASD. Web searches related to ASD and marijuana had an initial rise in 2009, coinciding with the commercialization of the legalized use of marijuana in some jurisdictions, and continue to exhibit an overall rising trend in web search popularity. Apart from searches about ASD and ABA (which have consistently been high), marijuana and ASD have been searched much more frequently than all other ASD intervention search terms evaluated since 2013.

The use of medicinal marijuana as a treatment option for children with ASD continues to be a topic of ongoing investigation. There is still a paucity of research and evidence in this area. While research is emerging on the feasibility and possible therapeutic applications [50,51], most research has been limited to observational and retrospective research. At present, there are no known published randomized controlled trials to evaluate the effectiveness, safety, and side-effect profile of the use of medicinal marijuana in children with ASD, or research providing information on their short- and long-term effects, but research is emerging. Autism Speaks, an ASD organization dedicated to advocacy and promotion of solutions for individuals with ASD, recently hosted a scientific consensus conference [52] on identifying the next steps in research on cannabis and autism with the goal of researching safety and potential benefits and developing expert consensus statements on this topic.

### ASD and Stem Cell Therapy

Stem cells have regenerative properties, and therefore have potential application in neurological disease. Their application in ASD has been proposed for their potential restorative and immunomodulatory properties that some researchers theorize could lead to better outcomes in children with ASD [53-55]. While theoretical concepts have led to proposals that stem cells may have the potential to be a promising therapy for ASD in the future, there are limitations, and further rigorous research is warranted [55]. Despite the publication of theoretical papers on stem cell therapy applied to ASD, public web search interest on this topic remains low. If future research yields positive promising results and especially if these are highlighted in the media, web search interest on this topic may increase.

### ASD and Fidget Spinners

Fidget spinners became popular among children, with a market sales peak in May 2017, accounting for 17% of all online toy sales [56]. At that time, there were considerable web-based claims of their benefits for children with ASD. However, a review of the research concluded that these alleged benefits have not been scientifically proven and that pediatricians should inform parents that these assertions have not been supported by peer-reviewed studies [57]. Not surprisingly, this study showed a surge in the web-based search frequency for fidget spinners and ASD in 2017 during the fidget spinner toy craze, followed by a marked drop in web-based search interest over the past 2 years, and the search term is no longer trending as an area of web-based search interest.

### Clinical Applications of Infodemiology Data for ASD

The web is often the first place people look for health information, likely because a search engine is usually more readily accessible [1,3] but it is also possible that some people may feel more comfortable to ask health questions to a search engine than a health professional and/or would like more information. The information from Google Trends analyses can therefore be used to help guide clinicians to open up discussions and create a safe space to ask those questions and enter into a dialogue providing expert, up-to-date, evidence-based information that is specific to a child’s unique needs.

The results of this study can be used to guide clinical discussions with parents of children already diagnosed with ASD or receiving a new diagnosis of ASD. This study highlights the fact that people are interested in knowing whether vaccines are associated with ASD, and discussing the research evidence and dispelling any myths surrounding this should be included in counselling when providing a diagnosis of ASD. Further, discussing what is currently known about the genetics of ASD is an area to highlight in counselling parents about etiological factors. Further, there is a great deal of web-based search interest in ABA as a treatment for ASD, and this should be discussed in detail with the parents and caregivers of children with a diagnosis of ASD in order to answer their questions about this intervention and other interventions they may have questions about.

Physicians caring for children with a diagnosis of ASD should always include discussions surrounding nutrition as part of each health visit. They should identify specific nutritional needs and symptoms, answer nutrition questions, and include evidence-based information about nutrition-based treatments for ASD and whether using those nutrition strategies are right and safe for the child’s specific nutrition needs. As highlighted in this study, different nutrition treatments have become more and less frequently searched on the web over time. Each child’s nutrition, health, and developmental needs are unique, and therefore, advice surrounding this should be part of the health supervision of children with ASD.

As discussed above, there were a number of search topics assessed in this study that still require more rigorous research. Clinicians working with children having ASD should stay abreast of new research evidence and recommendations on such topics. In particular, apart from searches relating to the ABA intervention, searches about the use of marijuana/cannabis in children with ASD have recently been performed more than other web searches for other interventions studied, with an overall upward trend. Research into marijuana/cannabis, the gut microbiome, probiotics, and stem cell therapy may yield more information in the future, and clinicians should be up to date on the research findings in order to answer clinical questions and provide evidence-based recommendations.

### Applications of Infodemiology and Infoveillance for Web-Based Knowledge Translation and Education Strategies, and the Use of Microdata Tagging for Search Engine Optimization

The results of this study demonstrate that Google Trends is an effective way to analyze data surrounding public interest of web-based information related to ASD over time and to observe trends in search queries. ASD advocacy organizations who aim to provide up-to-date and topical web-based information about ASD can use such data to monitor public web-based queries about ASD and can use this information to help guide web-based education initiatives. For example, in circumstances such as in 2017, when web searches about fidget spinners and ASD were trending, ASD organizations that provide informational content on their websites could have used such infoveillance data to identify the rise in public queries and guide content on their websites about this topic in order to provide accurate, up-to-date, and evidence-based information on this topic.

ASD and health or pediatric organization webmasters may use the data from this research and Google Trends analyses to adopt more sophisticated website search engine optimization techniques, such as microdata tagging, for helping guide web searches to land on evidence-based information and recommendations. Microdata tagging helps to tell search engines what the components of a webpage are, which can then be highlighted by search engines in web-based search engine queries. For example, ASD and vaccines are highly searched, whereas ASD and genetics are not commonly searched, but they represent important information when discussing the etiology of ASD. Microdata tagging can be used to guide searches about ASD and vaccines to content with evidence-based information about what is known about the etiology of ASD.

### Limitations

This study has some limitations that deserve comment. First, Google Trends normalizes data that are represented in a relative scaled score ranging from 0 to 100; however, the total search volume cannot be quantified. Second, these analyses are only able to represent web users and therefore exclude the ability to analyze information from those who do not have internet access, which may bias the results by excluding groups with limited internet access (eg, those with restricted resources and the elderly). Further, it does not capture web-based searches on other search engines; thus, it is not exhaustive. In addition, there are various ways in which this study could have been conducted, and while the search terms analyzed are comprehensive, they are not exhaustive. It should also be mentioned that autism could have been analyzed as either a search term or a topic and the data could have been analyzed specifying the health category or no specified category. Finally, there were no regional differences in web search patterns observed and therefore, maps of regional web searches were not presented in this study. Despite these aforementioned limitations, our results revealed public web-based search patterns in the field of ASD based on large population-based data collection, revealing information over 15 years that is easily accessible and reproducible, with a broad scope.

### Conclusions

Using Google Trends is an effective strategy for analyzing infodemiological trends in large-scale web search data about ASD. Analyses of web-based searches related to the causes and treatments of ASD showed variation over the past 15 years. These data provide valuable information that can be used to inform clinicians, and ASD and pediatric health organizations to help guide clinical discussions, web-based content, and education and knowledge translation strategies in order to provide up-to-date and evidence-based information and improve public, parent/caregiver, and patient awareness about ASD.

## Abbreviations

ABA: applied behavior analysis
ASD: autism spectrum disorder
MMR: measles, mumps, and rubella
